# Gene-based Therapy in a Mouse Model of Blue Cone Monochromacy

**DOI:** 10.1038/s41598-017-06982-7

**Published:** 2017-07-27

**Authors:** Yuxin Zhang, Wen-Tao Deng, Wei Du, Ping Zhu, Jie Li, Fan Xu, Jingfen Sun, Cecilia D. Gerstner, Wolfgang Baehr, Sanford L. Boye, Chen Zhao, William W. Hauswirth, Ji-jing Pang

**Affiliations:** 1 Ophthalmology, University of Florida, Gainesville, FL USA; 2 Department of Ophthalmology, First Affiliated Hospital, Nanjing Medical University, Nanjing, Jiangsu China; 3 Department of Ophthalmology, People’s Hospital of Guangxi Zhuang Autonomous Region, Nanning, Guangxi China; 4 Opthalmology and Visual Sciences, University of Utah, Salt Lake City, UT USA; 5 Department of Ophthalmology and Vision Science, Eye & ENT Hospital, Shanghai Medical College, Fudan University, Shanghai, China; 6 Xiamen Eye Center of Xiamen University, Xiamen, Fujian China; 7 Present Address: Ophthalmology Department of Peking University People’s Hospital, Peking University People’s Eye Center and Eye Institute, Beijing, China; 8 Present Address: Department of Obstetrics and Gynecology, Shanxi Dayi Hospital, Taiyuan, Shanxi Province China

## Abstract

Cones are responsible for daylight, central, high acuity and color vision. Three proteins found in human cones, i.e. long-wavelength (L)-, middle-wavelength (M)-, and short-wavelength sensitive (S)-opsins, are responsible for red, green and blue color recognition, respectively. Human blue cone monochromacy (BCM) is characterized by functional loss of both L- and M-cone opsins due to mutations in the *OPN1LW/OPN1MW* gene cluster on the X chromosome. BCM patients, who rely on their vision from only S-cones and rods, suffer severely reduced visual acuity and impaired color vision. Recent studies show that there is sufficient cone structure remaining in the central fovea of BCM patients to consider AAV-mediated gene augmentation therapy. In contrast, mouse retina has only two opsins, S-opsin and M-opsin, but no L-opsin. We generated an M-opsin knockout mouse (*Opn1mw*^−/−^) expressing only S-opsin as a model for human BCM. We show that recombinant M-opsin delivered by AAV5 vectors rescues M-cone function in *Opn1mw*^−/−^ mice. We also show that AAV delivered M-opsin localizes in the dorsal cone outer segments, and co-localizes with S-opsin in the ventral retina. Our study demonstrates that cones without M-opsin remain viable and respond to gene augmentation therapy, thereby providing proof-of-concept for cone function restoration in BCM patients.

## Introduction

Blue cone monochromacy (BCM) is an X-linked congenital disorder with severe cone dysfunction due to the absence of both long (*OPN1LW)* and medium (*OPN1MW)* wavelength-sensitive cone functions^1^. BCM patients display markedly reduced central vision, severely impaired color vision, photophobia (bright light sensitivity), and congenital nystagmus^2^. In humans, the *OPN1LW* gene and multiple copies of the downstream *OPN1MW* gene are arranged in a tandem 5′ to 3′ orientation^3,4^ on the X- chromosome. Yet, studies have shown that only the two most proximal genes in the cluster are expressed at an appreciable level^5,6^. The close proximity and high sequence homology^5^ subject the red and green pigment genes to unequal homologous recombination that can result in gene deletions, duplications, or hybrid genes^7–9^. Expression of these genes is regulated by a locus control region (LCR), a conserved *cis*-regulatory sequence located upstream of the *OPN1LW/MW* gene cluster^10^. BCM is frequently caused either by loss of this LCR or by harmful mutations within both genes, either of which will lead to the absence of both functional L- and M-cone opsins^1,11^. The two most common genetic causes of BCM are deletion of the LCR preventing the expression of the *OPN1LW/MW*, or the presence of either a single *OPN1LW/MW* hybrid opsin gene or multiple *OPN1LW/MW* genes with the most frequent deleterious missense mutation being a C203R substitution^1,12,13^.

Recent studies of BCM patients with Adaptive Optics Scanning Laser Ophthalmoscopy (AOSLO) confirm the potential for gene augmentation therapy. BCM retinas have variable degrees of a disrupted foveal cone mosaic and reduced cone density caused by degeneration of some L- and M-cones^14^. However, BCM foveal cone cell bodies were still present with detectable but rudimentary outer segments, potentially sufficient structural preservation for gene therapy^15,16^. Although BCM patients may show a slowly progressive cone degeneration with age^2,16^, patients with LCR deletions typically have a stationary, relatively non-progressive or very slowly progressive vision loss phenotype^17^. Identification of residual cone structure in the fovea and its slowly degenerative nature make BCM cones potentially viable targets for gene therapy^14–16^.

Significant progress has been made in understanding the molecular genetics of cone opsin deficiencies^18,19^. Studies focused on establishing the range of visual function and efficacy outcome measures for a BCM clinical trial have also been initiated^20,21^. However, animal models and the experimental literature on congenital cone opsin deficiencies are presently limited. In contrast to trichromatic humans, there are only two cone opsins in the mouse retina, S- and M-opsin. Most mouse cones express both opsins with a dorsal-ventral gradient in which M-opsin is expressed predominately in the dorsal retina and S-opsin mainly in the ventral retina^22–24^.

In this study, we tested whether AAV-mediated gene therapy can restore middle-wavelength cone function in a mouse model carrying a targeted deletion of the M-opsin gene, the *Opn1mw*^−/−^ mouse. This model was generated by inserting a gene trap into intron 2 of the *Opn1mw* gene. M-opsin is undetectable in *Opn1mw*^−/−^ retinal sections and by immunoblot. Middle-wavelength-mediated cone ERG signals are absent. The S-opsin dorsal/ventral distribution in this mutant strain is similar to that in wild type C57BL/6 J mice, establishing the *Opn1mw*^−/−^ mouse as a model for human BCM.

## Results

### M-opsin/S-opsin gradients in WT and *Opn1mw*^−/−^ retinas

**Figure 1:**
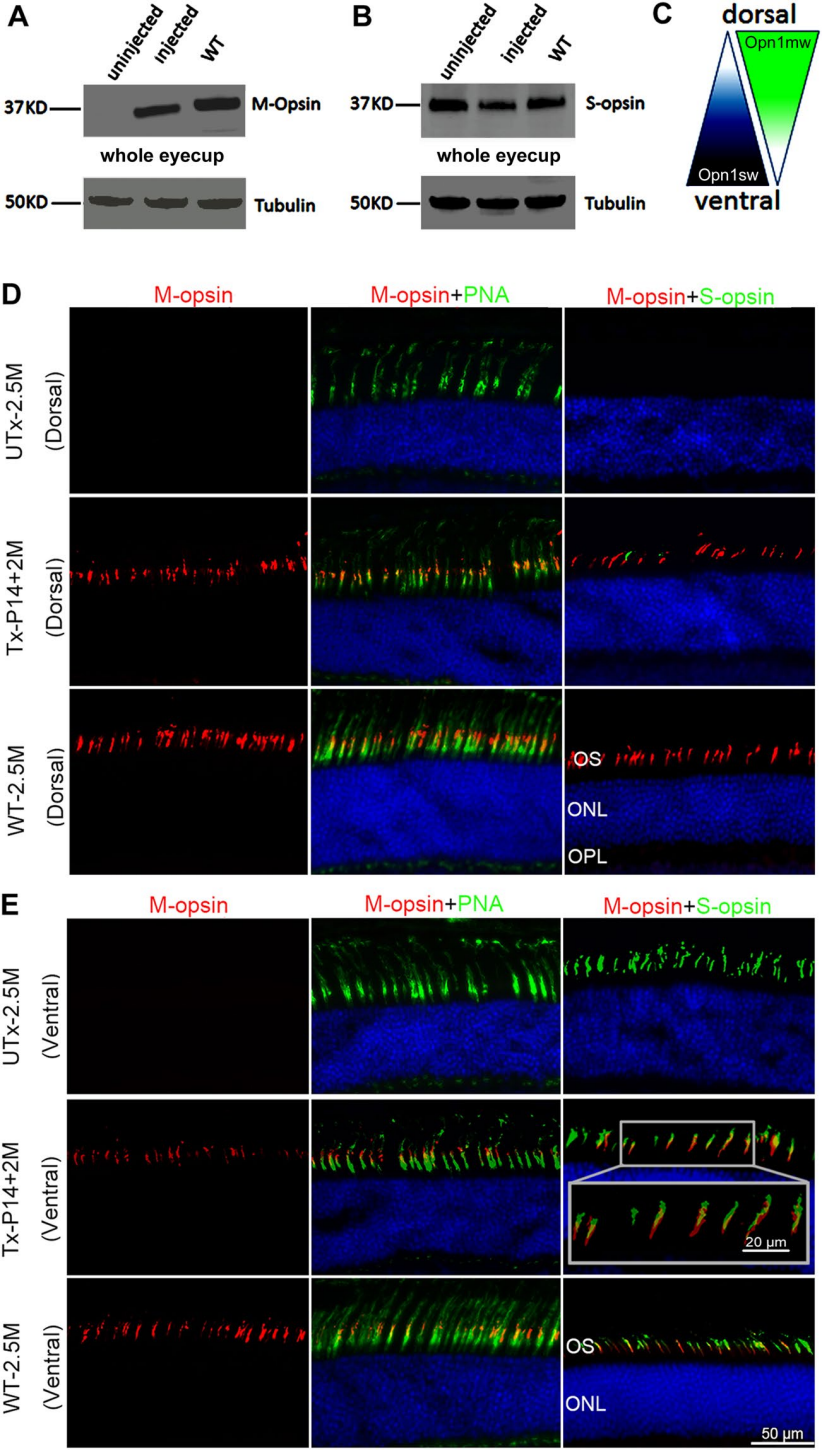
Comparison of M- and S-opsin in *Opn1mw*^−/−^ and WT mice. P14 *Opn1mw*^−/−^ mice were treated with AAV5-PR2.1-mouse M-opsin vector and western blot analysis of mouse M-opsin (**A**) and S-opsin (**B**) was performed at 2 month post-injection with age-matched wild type eyes (WT); Tubulin was served as loading control. Diagram of M- and S-opsin distribution in WT retina was shown in (**C**). Frozen sections were prepared for immunostaining from dorsal (**D**) and ventral (**E**) retina of 2.5-month-old untreated *Opn1mw*^−/−^ mice (UTx-2.5M), *Opn1mw*^−/−^ mice treated at P14 and analyzed at 2 months post-injection (Tx-P14 + 2 M), and age-matched wild type control mice (WT-2.5M). (**D**) Analysis of the dorsal retina with M- and S-opsin antibodies and PNA. Neither M- nor S-opsin was detected in the dorsal retinas of the untreated eye (UTx-2.5M), but PNA staining was normal. In the treated eye (Tx-P14 + 2 M), AAV-delivered M-opsin was now abundant in the dorsal retina and co-localized with PNA. In an untreated age-matched wild type eye (WT-2.5M), M-opsin is found co-localized with PNA with very little or no S-opsin in the dorsal retina. (**E**) Analysis of the ventral retina. S-opsin expression and PNA staining were normal in the untreated *Opn1mw*^−/−^ ventral retina (UTx-2.5M). AAV-delivered M-opsin co-localized with S-opsin (high magnification image is highlighted with a box) and PNA in cone outer segments in the treated *Opn1mw*^−/−^ ventral retina (Tx-P14 + 2 M). In the untreated wild type ventral retina (UTx-2.5), M-opsin is expressed in the ventral retina at a lower level compared to that in the dorsal retina but is detectable and is co-localized with PNA and S-opsin. 50μm in each figure.

*Opn1mw* germline knockout mice were generated using a cell line carrying a gene trapped (GT) *Opn1mw* gene (Supplementary Fig. S1). The gene trap was located in intron 2 (supplementary Fig. S1A) truncating OPN1MW after exon 2. Breeding *Opn1mw*^+/GT−^ mice produced female *Opn1mw*^*GT/GT−*^ and male *Opn1mw*^*Y/GT*^ animals at Mendelian ratios. Absence of M-opsin expression in *Opn1mw*^*GT/GT−*^ mice and presence of S-opsin was confirmed by western blot analysis (Fig. 1A,B).

**Figure 2:**
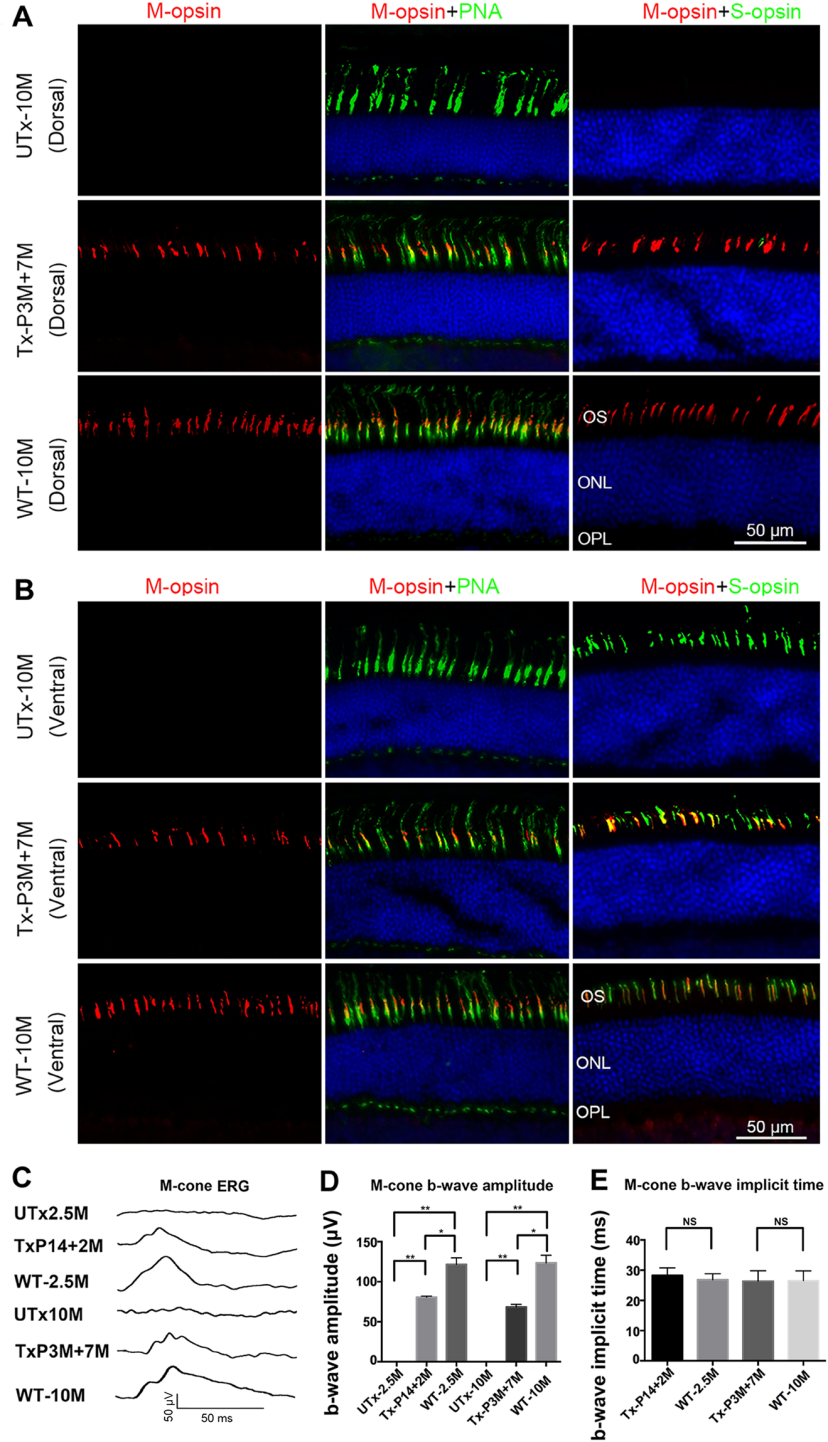
ERG analysis of middle wavelength cone function in mouse M-opsin treated *Opn1mw*^−/−^ eyes and immunostaining of retinal sections from 10-month-old untreated *Opn1mw*^−/−^ mice (UTx-10M), *Opn1mw*^−/−^ mice treated at 3 months of age and analyzed 7 months post-injection (Tx-P3M + 7M), and age-matched wild type uninjected control mice (WT-10M). (**A**) Analysis of dorsal retinas. M- and S-opsin were not detected in the dorsal retinas in the untreated eye (UTx-10M), whereas PNA staining is normal. AAV-delivered M-opsin was co-localized with PNA in the treated dorsal retina (Tx-P3M + 7M). An age matched wild type retina (WT-10M) showed normal M-opsin expression and undetectable S-opsin expression in the dorsal retina. (**B**) Analysis of ventral retinas. S-opsin expression and PNA staining were normal in the untreated *Opn1mw*^−/−^ ventral retina (UTx-10M). AAV-delivered M-opsin was co-localized with S-opsin and PNA in the treated *Opn1mw*^−/−^ ventral retina (Tx-P3M + 7M). An age-matched wild type eye (WT-10M) showed normal S-opsin expression and a detectable level of M-opsin expression in the ventral retina. (**C**) Representative middle wavelength ERG recordings from UTx-2.5M: an untreated 2.5 month old *Opn1mw*^−/−^ eye, TxP14 + 2 M: an *Opn1mw*^−/−^ eye treated at P14 with ERG analysis at 2 months post-treatment, UTx-10M: an uninjected wild type eye at 10 months of age, TxP3M + 7 M: an *Opn1mw*^−/−^ eye treated at 3 months of age with ERG analysis at 7 months post-treatment, and WT-10M: an uninjected wild type eye at 10 months of age. (**D**) Average b-wave amplitudes of middle wavelength ERGs from the above mice (n = 4 in each group), **P < 0.005, *P < 0.05, NS: no statistical differences. (**E**) Average b-wave implicit times of middle wavelength ERGs from above mice (n = 4). Untreated *Opn1mw*^−/−^ eyes were not included here since they have no detectable M-cone ERG response. NS: no statistical differences. Data are represented at as mean ± SD. Bar = 50μm in each figure.

In wild type mouse retinas, S-opsins distribute in a dorsal to ventral and M-opsins in a ventral to dorsal gradient (Fig. 1C). The characteristic dorsal-ventral gradients with M-opsin dominant in the dorsal retina and S-opsin dominant in the ventral retina were confirmed by immunohistochemistry (Fig. 1D,E). M-opsin was reduced but still detectable in the ventral retina (Fig. 1E WT-2.5M, left column), whereas S-opsin was absent in the distal end of dorsal retina (Fig. 1D WT-2.5M, right column), consistent with previous reports in C56BL/6 mice^22,25^. M-opsin was absent in both dorsal and ventral retinas of 2.5 and 10 month old untreated *Opn1mw*^−/−^ eyes (Fig. 1D,E UTx-2.5M, Fig. 2A,B UTx-10M, left columns). S-opsin expression in 2.5 and 10 month old untreated dorsal and ventral *Opn1mw*^−/−^ retinas was comparable to that seen in age-matched wild type retinas (Fig. 1 UTx-2.5M, Fig. 2A,B UTx-10M, right columns) suggesting that *Opn1mw*^−/−^ cones expressing only S-opsin do not degenerate at these ages.

### Viability of *Opn1mw*^−/−^ cones

To assess cone viability in the dorsal *Opn1mw*^*−/−*^ retina where M-opsin normally dominates in cone outer segments of wild type retinas, we labeled retinal sections with peanut agglutinin (PNA). PNA binds to the extracellular glycoprotein matrix of cone sheath that is secreted by the inner segment of cones and is therefore a marker of cone structural viability even in the absence of cone outer segment integrity^26^. In *Opn1mw*^−/−^ retinas, robust PNA labeling was observed in both dorsal and ventral retinas in 2.5 and 10 month old *Opn1mw*^−/−^ mice, suggesting that dorsal cones with little opsin may be viable and have survived for at least 10 months (Fig. 1D, UTx-2.5M and Fig. 2, UTx-10M, middle columns). Normal PNA-positive cone OS sheaths in the dorsal *Opn1mw*^−/−^ cones that contain very little if any S-opsin and no M-opsin, suggest the outer segments of 3M untreated *Opn1mw*^−/−^ cones can be replenished with M-opsin restoration.

### AAV5-mediated M-opsin expression in Opn1mw^−/−^ retinas

*Opn1mw*^*−/−*^ mice were injected subretinally in one eye with an AAV5 vector expressing mouse M-opsin driven by a cone-specific promoter PR2.1^27^, while contralateral eyes were untreated and served as controls. One set of mice were injected at postnatal day 14 (P14) and analyzed 2 months after injection (P14 + 2M). Western blot analysis showed that no M-opsin was detected in 2.5 month old untreated *Opn1mw*^−/−^ eyes, whereas vector treatment in P14 + 2M eyes led to about 60% of age-matched wild type M-opsin levels (Fig. 1A). We also analyzed S-opsin levels in these retinas (Fig. 1B). Following quantification of the protein in each lane and calculating the amount of S-opsin after normalizing to the tubulin, the S-opsin levels in the treated retinas and the wild type controls are about 70% and 74%, respectively, of that in untreated *Opn1mw*^−/−^ retinas. The slightly higher S-opsin level in untreated *Opn1mw*^−/−^ retinas compared to wild type is consistent with a previous study showing that M-opsin is upregulated in an S-opsin knockout mouse due to elimination of S-opsin mRNA competition^28^.

In these treated eyes, AAV-delivered M-opsin was detected in both dorsal and ventral retinas. In the distal end of the dorsal retina, M-opsin co-localized with PNA (Fig. 1D, Tx-P14 + 2M, middle column) suggesting PNA labeled cone sheaths in close proximity with outer segment discs loaded with recombinant M-opsin. Specifically, M-opsin was detected at the distal end of the cone sheaths after vector treatment (Fig. 1D,E, Tx-P14 + 2M, middle columns), suggesting the existence of normal cone outer segment discs. In the *Opn1mw*^−/−^ ventral outer segments where S-opsin normally dominates, AAV-delivered M-opsin co-localized with S-opsin and PNA to varying degrees (Fig. 1E, Tx-P14 + 2M, middle and right columns).

To test whether *Opn1mw*^−/−^ cones are also treatable in older animals, we injected 3-month-old *Opn1mw*^−/−^ mice subretinally as described above and analyzed them 7 months later (P3M + 7M mice). Immunostaining of retinal sections showed M-opsin expression again in both dorsal and ventral retinas (Fig. 2B) in a pattern similar to that seen in P14 + 2M retinas in both dorsal (Fig. 2A) and ventral hemispheres (Fig. 2B). These results suggest that treatment can be initiated in adult mice as old as 3 months and vectored M-opsin expression persists for an extended period of time (>seven months) after vector treatment.

### AAV5 Treatment of Opn1mw^−/−^ cones restores middle-wavelength ERG Responses

ERG responses to middle wavelength (510 nm) light stimuli were unrecordable in untreated *Opn1mw*^−/−^ eyes, consistent with the lack of functional M-opsin (Fig. 2C,D). The b-wave amplitudes of rod mediated scotopic ERGs and short wavelength mediated ERGs (short wavelength, 360 nm) in both 2.5 and 10 month old untreated *Opn1mw*^−/−^ eyes were similar to those seen in wild type eyes (Fig. S2). P14 + 2M treated eyes showed robust ERG restoration to middle wavelength photopic light flashes with an average b-wave amplitude of 73.78 ± 13.70 (average ± SD) at a flash light intensity of 25 cd-s/m^2^ (n = 4). This is 63% of the response seen in age-matched wild type controls (117.2 ± 11.26 μV, n = 4, P < 0.05). ERGs performed on the contralateral untreated eyes (n = 4, P < 0.005) were negative. P3M + 7M treated eyes showed similar levels of b-wave ERG restoration with an average amplitude of 62.95 ± 11.27 μV, which is 52% of the response seen in age-matched wild type controls (121.9 ± 8.448 μV, n = 4, P < 0.05) (Fig. 2C,D). The b-wave implicit times of the restored middle-wavelength ERGs were also normal in all treatment groups. Statistical analysis showed no difference in b-wave implicit times among P14 + 2M (28.68 ± 2.224 ms), wild type-2.5 M (27.93 ± 2.648), P3M + 7M (27.05 ± 3.078 ms) treated *Opn1mw*^−/−^ and wild type (27.43 ± 3.268 ms) eyes (Fig. 2E).

The average b-wave amplitudes of scotopic ERG responses were 458.9 ± 66.39 μV in untreated 2.5 M *Opn1mw*^−/−^ eyes (n = 6), 393.4 ± 30.48 μV in P14 + 2M treated *Opn1mw*^−/−^ eyes (n = 6) and 476.4 ± 97.50 μV in age-matched 2.5 M *C57 BL/6J* eyes (n = 6); 439.9 ± 82.12 μV in untreated 10 M *Opn1mw*^−/−^ eyes (n = 6), 407.2 ± 70.20 μV in P3M + 7M treated *Opn1mw*^−/−^ eyes (n = 6) and 494.7 ± 106.10 μV in age-matched 10M *C57 Bl/6J* eyes (n = 6). The ERG analysis is shown in Supplementary Fig. S2A, 2B. Thus, there was no effect of treatment on rod mediated ERG responses, as expected.

The average b-wave amplitudes of short wavelength cone ERGs were 124.70 ± 30.23 μV in untreated 2.5 M *Opn1mw*^−/−^ eyes (n = 7), 116.10 ± 35.83 μV in P14 + 2M treated *Opn1mw*^−/−^ eyes (n = 7) and 100.00 ± 33.30 μV in age-matched 2.5M *C57 BL/6J* eyes (n = 12); 110.00 ± 10.49 μV in untreated 10 M *Opn1mw*^*−/−*^ eyes (n = 6), 108.00 ± 16.97 μV in P3M + 7M treated *Opn1mw*^−/−^ eyes (n = 6) and 94.58 ± 26.84 μV in age-matched 10M *C57 Bl/6J* eyes (n = 12) (Figs 2D and S2C). Hence, like for rod function, there was also no effect of treatment on the pre-existing short wavelength-cone function. Although the average short wavelength ERG b-wave amplitudes was higher in both 2.5 M and 10 M *Opn1mw*^−/−^ eyes compared to that in age-matched wild type controls, statistical analysis suggested no difference.

## Discussion

The goal of this study was to test gene replacement retinal therapy in an *Opn1mw*^−/−^ mouse with only S-opsin as a model for treating human blue cone monochromacy. Untreated *Opn1mw*^−/−^ retinas appeared to have slightly higher S-opsin levels than their contralateral subretinally injected retinas and the wild type controls. A possible interpretation is that the S-opsin decrease in treated eyes may be due to damage related to subretinal vector induced transient retinal detachment. Subretinal injections of either AAV5-GFP or buffer as controls did not restore retinal functions as expected. However, all subretinal injections in mice induce retinal detachments and cause some degree of damage including outer segment shortening at the injection site. For this reason we use the uninjected eye but not AAV5-GFP or Buffer injected eye as the control.

The dorsal retina of *Opn1mw*^−/−^ mice partially mimics the fovea of BCM patients in terms of lacking detectible cone opsins. We demonstrate here that AAV-mediated expression of mouse M-opsin can restore ERG responses to middle wavelength light stimuli in *Opn1mw*^−/−^ mice. Moreover, recombinant mouse M-opsin was found in cone outer segments in both dorsal and ventral retinas. In the dorsal hemisphere where very little or no S-opsin is detectable, recombinant M-opsin localized at the distal ends of cone outer segments surrounded with PNA, a marker of cone outer segment sheaths. In the ventral hemisphere where S-opsin pre-exists, recombinant M-opsin co-localized with endogenous S-opsin in a ratio that varied from cell to cell, but without discernable effect on the 360 nm ERG. These results demonstrate that even without M-opsin and very low S-opsin, dorsal cones remain viable and can be targeted for successful gene therapy. Importantly, cones with one type of cone opsin, S-opsin, (ventral *Opn1mw*^−/−^ cones) can reform normal cones upon delivery of recombinant M-opsin without physiological effects. This observation provides proof-of-concept for treatment of BCM patients and perhaps also for X-linked red/green color blindness (protan or deutan deficits).

There are three types of cone opsins, M, L and S-opsin in the human retina. Cones consisting L and M opsins are the most abundant and comprising about 95% of the total cone population with their peak density in the central fovea^29,30^. The M/L-cone rich fovea, particularly the M/L-cone only central foveola, is the principle reason why BCM patients who are missing M- and L-opsins have not only color vision problems, but, more critically, also have impaired visual acuity. Since cone opsins are the primary component of the cone outer segment disk membrane, identification of residual inner cone structure preservation in BCM fovea suggests that these cones can survive without opsin, an indication of their potential for responding to gene therapy intervention^14–16^. Our results in the *Opn1mw*^−/−^ mice confirm that distal end of the dorsal cones have indeed survived despite the absence of any opsins including M-opsin. This is consistent with the earlier observation in an S-opsin knockout mouse that cones in the distal ventral retina lacking M-opsin with minimal or no-opsin can survive for at least 1.5 years^28^. The demonstration that cones without opsin remain viable and have the ability to respond to gene augmentation therapy provides animal proof-of-concept for treating BCM patient cones which have shortened or vestigial cone outer segments.

In addition to the presence of residual cone structure in patients, another independent positive development for BCM gene therapy is the previous demonstration of successful gene therapy in adult dichromatic monkeys missing the L-opsin gene (protanopia) and therefore red colorblind since birth^31^. Restoration of trichromatic visual behavior in these monkeys when an AAV vectored human L-opsin was expressed in foveal cones suggested that the addition of a novel cone function in an adult nonhuman primate leads to the development of new cone inputs to the mature visual cortex, and that this mediates improved color perception and color vision elicited behavior even well after early development.

In normal C57 mice, about 40% of cones express both M- and S-opsins, and 26% cones only express the S-opsin. This means more than half of the cones express S-opsin in normal pigmented mice^23^. In human and primate retinas, cones with S-opsin compromise only 3–5% of foveal cones, which distribute in a gradient pattern with an ‘S-opsin free zone’ in the center of fovea^32^. Photophobia is typical in severe inherited human cone-related diseases like achromatopsia or BCM, but does not occur in mouse models. It is unknown whether lack of photophobia in *Opn1mw*^−/−^ mice is associated with higher S-opsin expression in this model. It is conceivable that increased S-cone numbers in Opn1mw^−/−^ mice is at least the partial reason for less severe cone degeneration in this strain.

In summary, our data suggest that dorsal cone outer segment that has little opsin still has the ability to regenerate normal outer segment discs following AAV-mediated M-opsin expression in an M-opsin knockout mouse. Demonstration of AAV delivered opsin expression in these cones and restoration of middle wavelength cone function in *Opn1mw*^−/−^ mice is clearly a promising proof-of-concept for eventually treating foveal cones in BCM patients.

## Materials and Methods

### Animals

All mice used in this study were maintained in the University of Florida Health Science Center Animal Care Service Facilities on a 12 hour/12 hour light/dark cycle. All animals were maintained under standard laboratory conditions (18 –23 °C, 40–65% humidity) with food and water available ad libitum. All experiments were approved by the Institutional Animal Care and Use Committee at the University of Florida and conducted in accordance with the ARVO Statement for the Use of Animals in Ophthalmic and Vision Research and National Institutes of Health regulations.

### Generation of Opn1mw knockout

An ES cell line carrying the *Opn1mw* gene into which a 7122 BP gene trap was inserted in intron 2 (International Knockout Mouse Consortium, EUCOMM project) was used to generate chimeras at the University of Utah transgenic core (Fig. S1)^33,34^. The gene trap consists of a splice acceptor sequence, an IRES (internal ribosome entry site) sequence, a promoterless lacZ gene, an SV40 termination signal, and a neomycin selection cassette (Fig. S1A). Proper location of the trap and the presence of 5′ and 3′ arms as well as the location of loxP in intron 3 were confirmed by PCR and sequencing (Fig. S1B and S1C). Animals were genotyped using primers GF6 (5′-TGGGTCCCTGAGGCTTGTGT), GR6 (5′-CCTCAACTTCAGCCTGCACACTTC) and LAR3B (5′-CAAGGGCAGTAGAGTTCCCAG) (Fig. S1D). The WT allele yields an amplicon of 350 bp; mutant allele, 600 bp. Chimeric mice were outbred to C57BL/6J mice to generate *Opn1mw*^+/−^ mice. Breeding *Opn1mw*^+/GT−^ mice produced *Opn1mw*^*GT/GT−*^ animals at Mendelian ratios.

### Construction of AAV vectors

The mouse *Opn1mw* cDNA was purchased from American Type Culture Collection (Manassas, VA). The cDNA was amplified by PCR with primers containing NotI sites at both ends: the forward primer was gcttatgcggccgccaccatggcccaaaggcttacag and reverse primer was gcttatgcggccgcttatgcaggtgacactgaag. The PCR fragments were cloned into an AAV vector plasmid containing the PR2.1 promoter which has been shown to target transgene expression to mammalian L/M cones^27^ and the sequence of the final construct confirmed for accuracy. All AAV vectors were packaged in serotype 5 and were purified according to previously published methods^35^.

### Subretinal vector injection

Trans-corneal subretinal injections were performed to either P14 or P90 *Opn1mw*^*−/−*^ eyes with a 33-gauge blunt needle mounted on a 5 ml Hamilton syringe. First, an entering hole was introduced at the edge of the cornea by a 30-gauge needle, then the Hamilton syringe loaded with 1 µl of vector mixed with fluorescein dye was guided through the corneal opening into the subretinal space by previously described methods with minor modification^36–38^. Fluorescein was added to the vector solution at a final concentration of 0.1% to allow visualization of the subretinal bleb. In animals with no apparent surgical complications, only those whose initial retinal blebs occupied more than 90% of the retina were retained for further evaluation. One percent atropine eye drops and neomycin/polymyxin B/dexamethasone ophthalmic ointment were applied after injection. One eye was injected while the contralateral eye served as an untreated control.

### Electroretinography

ERG recordings were performed with a UTAS Visual Diagnostic System with a Big Shot Ganzfeld dome (LKC Technologies, Gaithersburg, MD) using previously described methods with modifications^39,40^. All mice were anesthetized by intraperitoneal injection of ketamine (72 mg/kg)/xylazine (4 mg/kg). The pupils were dilated with 1% atropine and 2.5% phenylephrine hydrochloride. After overnight dark adaptation, scotopic ERGs were recorded at stimulus light intensity of 0.025 cd.s/m^2^ (−1.6 log cd.s/m^2^) with inter-stimulus intervals of 30 seconds, and 10 recordings averaged. Middle wavelength and short wavelength ERGs were recorded at 510 nm and 360 nm respectively after light exposure for 5 min at 30 cd/m^2^. M-cone ERGs were recorded at 25 cd.s/m^2^ (1.4 log cd.s/m^2^) stimulus intensities, and Short wavelength ERGs recorded at 2.5 cd.s/m^2^ (0.4 log cd.s/m^2^) stimulus intensities. Twenty-five recordings were averaged for each stimulus light intensity and ERG data presented as an average ± SD. Statistics were performed using the unpaired t-test and significance defined as a P value of less than 0.05.

### Tissue preparation and immunohistochemistry

Mice were sacrificed and their eyes marked at 12 o’clock on the cornea with a burn mark and enucleated immediately employing previously described methods^41–43^. The eyes were fixed in 4% paraformaldehyde overnight at 4°C, the cornea and lens then removed without disturbing the retina. For frozen retinal sections, the eyecups were rinsed with PBS and cryoprotected by 30% sucrose/PBS for 3 hours at room temperature, then embedded in cryostat compound (Tissue TEK OCT, Sakura Finetek USA, Inc., Torrance, CA) and frozen at −80 °C. Retinas were cut perpendicularly from dorsal to ventral at 12 μm thickness. For immunohistochemistry, retinal sections were rinsed in PBS and blocked in 2% normal goat serum, 0.3% Triton X-100, 1% BSA in PBS for 30 min at room temperature. Sections were then incubated with either lectin peanut agglutinin (PNA) conjugated with Alexa Fluor 488 (1: 200, L21409, Invitrogen), or M- or S- opsin primary antibodies (1:400, Millipore, Temecula, CA) diluted in 0.1% Triton and 1% BSA in PBS overnight at 4 °C. They were then washed with PBS three times, followed by incubation with IgG secondary antibody tagged with Alexa-594 or Alexa-488 (molecular Probes, Eugene OR) diluted 1:500 in PBS at room temperature for two hours followed by washing with PBS. Sections were mounted with Vectashield Mounting Medium for Fluorescence (H-1400, Vector lab, In. Burlingame, CA) and cover slipped. Whole mounts and sections were analyzed with a Zeiss CD25 microscope fitted with Axiovision Rel. 4.6 software.

### Western Blot Analysis

Untreated 2.5M, treated P14 + 2 M *Opn1mw*^−/−^ eyes and wild type 2.5M eyes (three eyes each) were carefully dissected, and the eyecups were pooled and homogenized by sonication in a buffer containing 0.23 M sucrose, 5 mmol/L Tris-HCl, pH 7.5, and protease inhibitors (Roche Complete). After centrifugation, aliquots of the retinal extracts containing equal amounts of protein (50 µg) were analyzed by electrophoresis on 4–15% polyacrylamide–SDS gels, transferred to Immobilon-FL membranes (Millipore), and probed with the same M- and S-opsin antibodies used for immunohischemistry. An antibody against α-tubulin (rabbit polyclonal ab4074; Abcam) was used as an internal loading control. Visualization and quantification of specific bands was performed using the Odyssey Infrared Fluorescence Imaging System (Odyssey; Li-Cor).

### Data availability

Authors confirm that all relevant data are included in the article and its supplementary information files. The datasets generated and analyzed during the current study are available from the corresponding author on reasonable request.

## Electronic supplementary material

Supplementary Information
