# Publisher Correction: Gene-based Therapy in a Mouse Model of Blue Cone Monochromacy

**DOI:** 10.1038/s41598-018-23131-w

**Published:** 2018-03-14

**Authors:** Yuxin Zhang, Wen-Tao Deng, Wei Du, Ping Zhu, Jie Li, Fan Xu, Jingfen Sun, Cecilia D. Gerstner, Wolfgang Baehr, Sanford L. Boye, Chen Zhao, William W. Hauswirth, Ji-jing Pang

**Affiliations:** 1 Ophthalmology, University of Florida, Gainesville, FL USA; 2 Department of Ophthalmology, First Affiliated Hospital, Nanjing Medical University, Nanjing, Jiangsu China; 3 Department of Ophthalmology, People’s Hospital of Guangxi Zhuang Autonomous Region, Nanning, Guangxi China; 4 Opthalmology and Visual Sciences, University of Utah, Salt Lake City, UT USA; 5 Department of Ophthalmology and Vision Science, Eye & ENT Hospital, Shanghai Medical College, Fudan University, Shanghai, China; 6 Xiamen Eye Center of Xiamen University, Xiamen, Fujian China; 7 Present Address: Ophthalmology Department of Peking University People’s Hospital, Peking University People’s Eye Center and Eye Institute, Beijing, China; 8 Present Address: Department of Obstetrics and Gynecology, Shanxi Dayi Hospital, Taiyuan, Shanxi Province China

Correction to: *Scientific Reports* 10.1038/s41598-017-06982-7, published online 27 July 2017

The original version of this Article contained errors in the spelling of the author Sanford L. Boye, which was incorrectly given as L. Boye Sanford. This has now been corrected in both the PDF and HTML versions of the Article.

