# Supplementary material for: Gene-based Therapy in a Mouse Model of Blue Cone Monochromacy
Source: Sci Rep. 2017 Jul 27;7:6690. doi: 10.1038/s41598-017-06982-7 (PMC5532293; doi:10.1038/s41598-017-06982-7)
Supplement: Supplementary file 1 — Supplementary Information [file 41598_2017_6982_MOESM1_ESM.pdf]

Gene-based Therapy in a Mouse Model of Blue Cone Monochromacy

Yuxin Zhang[1,2], Wen-Tao Deng[1], Wei Du[1#], Ping Zhu[1], Jie Li[1], Fan Xu[1,3], Jingfen Sun[1##], Cecilia D. Gerstner[4], Wolfgang Baehr[4], Sanford, L. Boye[1], Chen Zhao[2,5*], William W. Hauswirth[1*], Ji-jing Pang[1,2,6*]

[1]Ophthalmology, University of Florida, Gainesville, FL. [2]Department of Ophthalmology, First Affiliated Hospital, Nanjing Medical University, Nanjing, Jiangsu, China. [3]Department of Ophthalmology, People's Hospital of Guangxi Zhuang Autonomous Region, Nanning, Guangxi, China. [4]Opthalmology and Visual Sciences, University of Utah, Salt Lake City, UT, [5]Department of Ophthalmology and Vision Science, Eye & ENT Hospital, Shanghai Medical College, Fudan University, Shanghai, China. [6]Xiamen Eye Center of Xiamen University, Xiamen, Fujian, China.

*Corresponding authors; correspondence should be addressed to Ji-jing Pang at Department of Ophthalmology, College of Medicine, University of Florida, 1600 SW Archer Road, Gainesville, FL 32610. Tel: 1-352-2739341; Fax: 1-352-3920573; Email: jpang@ufl.edu.

The authors wish it to be known that, in their opinion, the first 3 authors should be regarded as joint First Authors.

Current address: #: Ophthalmology Department of Peking University People's Hospital, Peking University People's Eye Center and Eye Institute, Beijing, China. ##: Department of Obstetrics and Gynecology, Shanxi Dayi Hospital, Taiyuan, Shanxi Province, China

Supplementary Figures and Legends

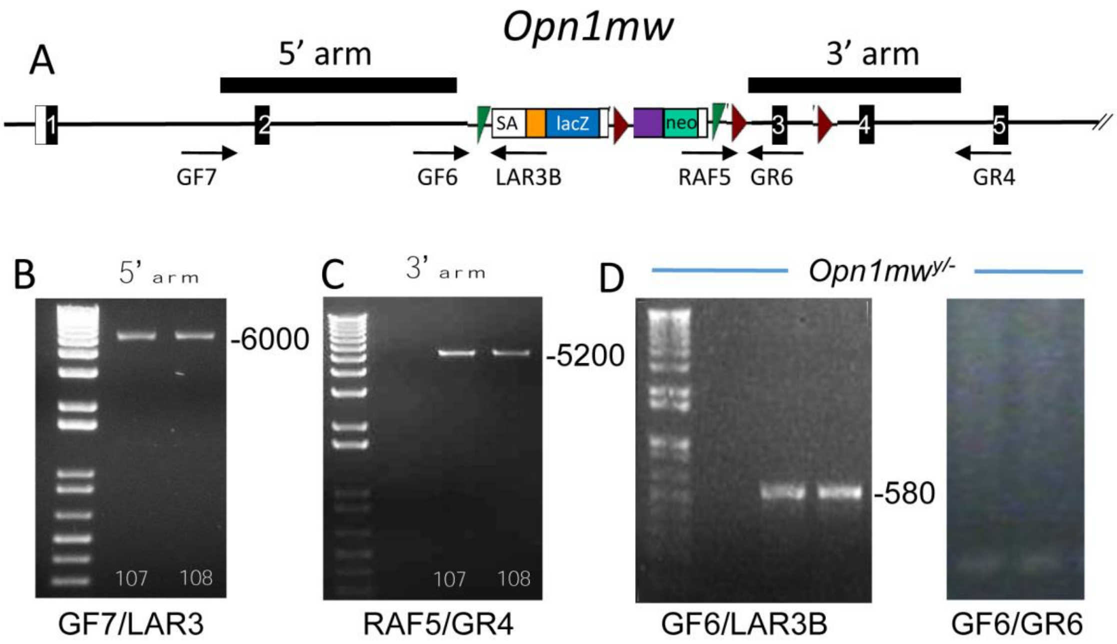

Supplementary Figure 1. Generation of Opn1mw knockout mouse. A, schematic of the Opn1mw gene with gene trap inserted into intron 2. Exons 1-5 are marked with black boxes. Approximate sites of 5' and 3' arms are indicated. SA: splice acceptor site; lacZ: promoterless β-galactosidase gene. Brown triangles: loxP signals; Green triangles: FRT signals; purple rectangle: SV40 polyA transcription termination; neo: neomycin cassette. B&C, verification of the presence of long and short arms in the gene trapped cell line. D, genotyping of an Opn1mw$^{-/-}$ mouse.

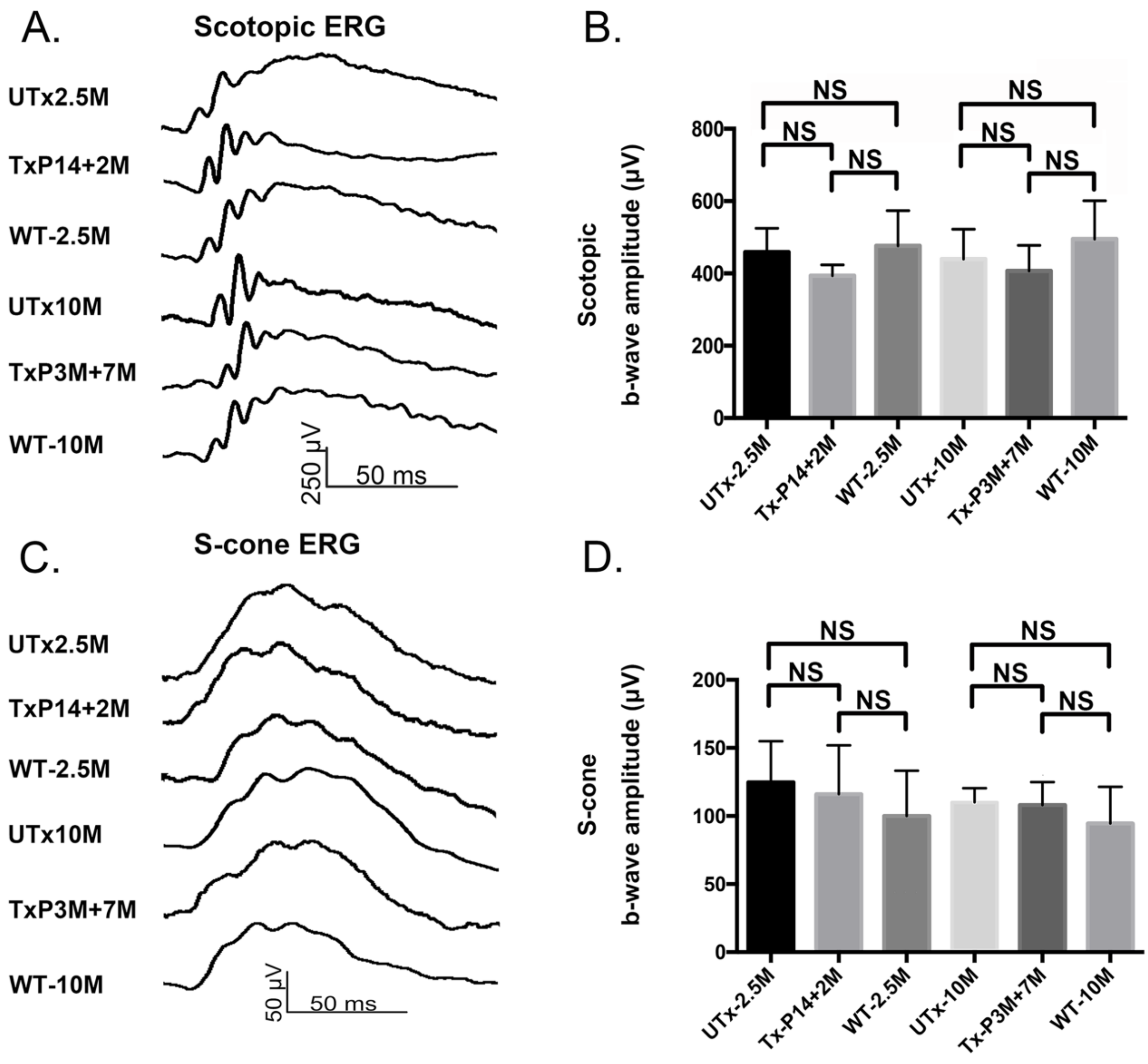

Supplementary Figure 2. Scotopic and short wavelength photopic ERG responses from untreated 2.5 month old *Opn1mw*$^{-/-}$ mice (UTx2.5M), *Opn1mw*$^{-/-}$ mice treated at P14 with ERG analysis at 2 months post-treatment (TxP14+2M), untreated 2.5-month-old wild type mice (WT-2.5M), untreated 10 month old *Opn1mw*$^{-/-}$ mice (UTx10M), *Opn1mw*$^{-/-}$ mice treated at 3 months of age with ERG analysis at 7 months post-treatment (TxP3M+7M), and untreated 10 month old wild type mice (WT-10M).

(A) Representative rod-mediated scotopic ERG recordings. (B) Average b-wave amplitudes of scotopic ERGs. (C) Representative short wavelength ERG recordings. (D) Average b-wave

amplitudes of short wavelength ERGs. NS: no statistical difference. Data are represented at as mean $\pm$ SD.
